# Feasibility, Acceptance, and Safety of Metacognitive Training for Problem and Pathological Gamblers (Gambling-MCT): A Pilot Study

**DOI:** 10.1007/s10899-020-09975-w

**Published:** 2020-09-21

**Authors:** Josefine Gehlenborg, Lara Bücker, Mira Berthold, Franziska Miegel, Steffen Moritz

**Affiliations:** Department of Psychiatry and Psychotherapy, University Medical Center Hamburg-Eppendorf, Martinistraße 52, 20246 Hamburg, Germany

**Keywords:** Gambling, Metacognition, Treatment, Feasibility, Acceptance, Safety

## Abstract

Approximately 90% of problem and pathological gamblers remain untreated. This treatment gap may be diminished by the implementation of low-threshold treatment programs. As cognitive distortions play a crucial role in the development and maintenance of gambling problems, interventions targeting gambling-related biases may be particularly effective. The aim of the present study was to examine the feasibility, acceptance, and safety of a novel metacognitive training for individuals with gambling problems (Gambling-MCT). Twenty-five participants were included in an uncontrolled pilot trial with two assessment points (intervention). The intervention comprised eight training modules targeting gambling-specific cognitive distortions. At baseline and post assessment, symptom severity, as measured with the PG-YBOCS, and gambling-related cognitive distortions, as measured with the GABS, were assessed. In addition, interim assessments measuring session-specific changes were conducted. Subjective appraisal was examined after each module and also post treatment. On average, participants took part in 4.16 (*SD* = 2.84) training sessions. Both intent-to-treat and per protocol analyses showed significant improvements on the PG-YBOCS and the GABS (*d*_*z*_ = 0.37–1.37). After Bonferroni correction for multiple testing, linear mixed models for the analysis of session-specific changes showed no deterioration in participants’ mental state after any of the modules. Subjective appraisal of Gambling-MCT was good. The present pilot study provides first evidence for the feasibility, acceptance, and safety of Gambling-MCT. Recruitment of participants remains challenging, emphasizing the importance of overcoming patient-related treatment barriers. Future studies need to investigate the efficacy of Gambling-MCT in randomized controlled trials.

**Electronic supplementary material:**

The online version of this article (10.1007/s10899-020-09975-w) contains supplementary material, which is available to authorized users.

## Introduction

Archaeological findings suggest that games of chance go back to approximately 4000 years BC (Hunt and Blaszczynski 2019). Over the centuries, gambling has become a common leisure activity, and it is estimated that more than 70% of people of legal age have participated in gambling activities at least once (Kessler et al. 2008; Meyer et al. 2011). Although gambling is in most cases unproblematic, a subgroup of gamblers develops addictive patterns of gambling behavior with prevalence rates of pathological gambling of 0.5% to 7.6% worldwide (Williams et al. 2012). In the fifth edition of the *Diagnostic and Statistical Manual of Mental Disorders* (*DSM-5*; American Psychiatric Association 2013), pathological gambling is classified as a behavioral addiction. For diagnosis, at least four of nine symptoms need to be present in a 12-month period (e.g., development of tolerance, loss of control, gambling to compensate for losses [“chasing”]). Pathological gambling results in severe psychosocial and financial problems (Cowlishaw et al. 2016; Gainsbury et al. 2014). But even problem gamblers not meeting *DSM-5* diagnosis criteria experience serious impairments in psychosocial functioning such as low life satisfaction and stressful life events compared to healthy individuals (Weinstock et al. 2017). Moreover, problematic gambling behavior represents one of the most relevant risk factors for the transition to pathological gambling (Dowling et al. 2017).

### Cognitive Distortions in Problem and Pathological Gambling

Individuals with gambling problems often display specific cognitive biases and beliefs (Ciccarelli et al. 2017; Fortune and Goodie 2012) that are associated with gambling severity (Cocker and Winstanley 2015). These cognitive distortions relate to the overestimation of one’s own influence in gambling as well as the chances of winning (Clark 2010) and contribute to the persistence of gambling behavior within a gambling session as well as the maintenance of pathological gambling between sessions (Sharpe 2002). Cognitive distortions increase the motivation to continue gambling (Clark et al. 2009; Ladouceur and Sévigny 2005; Stange et al. 2016, 2017) and have been identified as a predictor of treatment failure (Rossini-Dib et al. 2015) and relapse (Oei and Gordon 2008; Smith et al. 2015). The most frequently reported distortions implicated in the development and maintenance of pathological gambling are near misses, illusion of control, gambler’s fallacy, illusionary correlations (e.g., superstitious beliefs), and attributional and memory bias (Fortune and Goodie 2012; Hodgins et al. 2011). For an overview of empirical findings regarding cognitive distortions in problem and pathological gambling, see the electronic supplementary material (supplementary material A). The amelioration of cognitive biases may play a key role in the successful treatment of pathological gambling (Fortune and Goodie 2012; Gooding and Tarrier 2009; Tavares et al. 2003).

### Gambling and Depression

Almost 80% of problem gamblers and more than 95% of pathological gamblers show comorbid psychiatric disorders (Meyer et al. 2011), with particularly striking comorbidity rates of around 30% to 40% for depressive disorders (Dowling et al. 2015; Lorains et al. 2011; Meyer et al. 2011). In more than 70% of cases, depressive symptoms occur before the onset of gambling problems (Kessler et al. 2008) and may be a relevant risk factor for gambling problems (Dowling et al. 2017; Keough et al. 2018). According to the self-medication hypothesis (Khantzian 1997), depressed individuals gamble to escape negative emotions, which in turn increases the severity of their gambling problems (Bilevicius et al. 2018). At the same time, depressive symptoms may also occur in response to the financial burden of gambling, which further strains psychosocial functioning (Blaszczynski and Nower 2002). The reciprocal relationship between depressive symptoms and gambling disorder has been shown to be mediated by gambling-related distorted cognitions (Schluter et al. 2019). In addition, there is preliminary evidence that treating comorbid depressive symptoms in problem and pathological gamblers not only leads to a reduction in depressive symptoms but also reduces gambling symptoms (Bücker et al. 2018). Targeting comorbid depressive symptoms such as depressive cognitive distortions therefore represents an important aspect in the treatment of gambling problems.

### Treatment Gap for Problem and Pathological Gambling

Psychotherapy holds promise for the treatment of pathological gamblers (Ginley et al. 2019). Most treatment studies have examined elements of cognitive behavioral therapy (CBT), showing large effect sizes post treatment (Cowlishaw et al. 2012; Gooding and Tarrier 2009; Petry et al. 2017). Notwithstanding initial evidence for the efficacy at 6-, 12-, and 24-month follow-up (Gooding and Tarrier 2009), there is a dearth of long-term studies (Cowlishaw et al. 2012; Petry et al. 2017). Even though effective treatment approaches exist, approximately 90% of problem and pathological gamblers remain untreated (Meyer et al. 2011; Slutske 2006). Moreover, the treatment course of the small proportion of pathological gamblers who do seek treatment is characterized by high dropout rates (Melville et al. 2007; Ronzitti et al. 2017). This treatment gap is owing to a multitude of factors relating to both the individuals with gambling problems and structural aspects of the help system (Dąbrowska et al. 2017). With respect to the latter, the high rate of untreated pathological gamblers may be due to the unavailability and lack of treatment programs (Khayyat-Abuaita et al. 2015; Thaller et al. 2017), lack of awareness of treatment options, and financial aspects (Suurvali et al. 2009). On the other hand, individuals with pathological gambling tend to deny or downplay their problems and do not seek treatment until their problems have culminated in a major life crisis (Clarke et al. 2007). This behavior may be caused by the wish to cope with the problem by oneself due to shame as well as concerns about treatment (e.g., fear of failing in therapy, doubts about quality and efficacy of treatment) and practical treatment aspects (e.g., concerns about cost, lack of time; Dąbrowska et al. 2017; Khayyat-Abuaita et al. 2015; Suurvali et al. 2009). To overcome the treatment gap for pathological gambling, effective, low-cost, and low-threshold treatment programs need to be developed and disseminated on a large scale.

### Metacognitive Training

The term metacognition was coined by James H. Flavell at the end of the 1970s to signify “knowledge and cognition about cognitive phenomena” (Flavell 1979, p. 906). Flavell distinguished four components of metacognition: metacognitive knowledge, metacognitive experiences, metacognitive goals (tasks), and metacognitive actions (strategies). In brief, metacognition can be defined as thinking about one’s thinking. Metacognitive training (MCT; Moritz et al. 2014) aims to “straighten” distorted thought processes that may contribute to psychiatric disorders (Zhang et al. 2018). MCT is a group intervention that was first developed in the early 2000s for the treatment of individuals with psychosis. The aim of the intervention is to playfully uncover and change cognitive distortions that influence the perception, interpretation, and memory of information. In doing so, corrective experiences should result in metacognitive experiences (“aha moments”), which in turn can improve metacognitive knowledge, particularly knowledge about cognitive distortions (Moritz and Lysaker 2018). Over the years, MCT has been adapted to a variety of disorders, such as depression (D-MCT; Jelinek et al. 2015), borderline personality disorder (B-MCT; Schilling et al. 2018), and obsessive–compulsive disorder (MCT-OCD; Miegel et al. 2019). All versions of MCT pursue a normalizing treatment rationale. The training adopts a dialectical approach by emphasizing that biases are also prevalent in individuals not suffering from psychological disorders but that an escalation may foster psychological problems. Further, the training encompasses psychoeducational elements as well as established strategies from CBT and third wave techniques (e.g., mindfulness). MCT is a low-threshold treatment that is intended to be an “add-on” intervention used in addition to other treatment elements (e.g., individual psychotherapy, psychopharmacotherapy). Efficacy of MCT for psychosis has been shown in meta-analyses to have moderate effect sizes (*g* =  − 0.34 to − 0.41) for symptom reduction and strong effect sizes (*g* =  − 0.84) for acceptance of the intervention (Eichner and Berna 2016; Liu et al. 2018).

### Aim of the Present Study

The aim of the present study was to evaluate a new low-threshold treatment approach (Gambling-MCT) that targets gambling-specific cognitive distortions and comorbid depressive symptoms in a sample of problem and pathological gamblers. The study was conducted as an uncontrolled pilot trial (phase I) targeting feasibility, acceptance, and safety (i.e., the absence of negative side effects on symptomatology) of the intervention. Findings of the study will be used for further development of treatment materials and the manual.

## Methods

### Study Design

In order to evaluate the feasibility, acceptance, and safety of Gambling-MCT, we conducted an uncontrolled treatment study. Gambling-MCT was offered weekly in an open group format that allowed participants to join the group at any time. The intervention period (pre-post) was eight weeks. Symptom severity was measured at pre and post assessments. Moreover, interim assessments before and after each training session were conducted to assess the current mental state of participants in order to evaluate session-specific effects. At post assessment as well as after each module, we asked for subjective appraisal of the Gambling-MCT. The post survey was offered online to those who were not able to attend their last training session. To receive the link to the online survey using the software Unipark^®^ (EFS Survey), participants voluntarily provided an e-mail address on the consent form at baseline. After completing the post assessment, participants received a 15€ Amazon voucher as an incentive, irrespective of the number of group sessions attended. All data were assessed pseudonymously. Participants were instructed to create a personal code word prior to the baseline assessment, based on criteria suggested by the German Psychological Society (DGPs). The pilot study was approved by the Local Psychological Ethics Commission at the Center for Psychosocial Medicine (LPEK) at the University Medical Center Hamburg-Eppendorf (Germany, LPEK-004) and was conducted in accordance with the Declaration of Helsinki. As this project was conducted as an exploratory, open-ended pilot study, the trial was not preregistered.

### Participants

Gambling-MCT is targeted at both problem and pathological gamblers. Inclusion criteria for this study were (a) subjective gambling problems (no diagnosis was mandatory), (b) age of 18 to 70 years, (c) informed consent to participate in the study, (d) the ability and willingness to participate regularly in the eight-week training program, and (e) sufficient command of the German language. Lifetime diagnoses of schizophrenia or bipolar disorder or the presence of acute suicidal tendencies, assessed at baseline via self-report, represented exclusion criteria. Study participation was allowed if participants were undergoing other therapeutic interventions (e.g., psychopharmacotherapy or psychotherapy). Problem and pathological gamblers were recruited in an outpatient institution as well as a counseling center for addiction in Hamburg, Germany, from June 2018 to December 2019. Participants recruited from the counseling center were not necessarily treated in the facility as some were recruited from the facility’s wait list. In addition, flyers about the study were displayed in more than 80 gambling halls as well as counseling centers, and a Google^®^ AdWords campaign was initiated. The campaign was displayed to individuals in the region of Hamburg (Germany) who searched for relevant keywords (e.g., “group therapy” and “gambling”) using the search engine Google^®^.

### Questionnaires

#### Sociodemographic Questionnaire

At baseline, sociodemographic data were assessed, including gender, age, years of education, employment status, and nationality. In addition, participants were asked about psychiatric diagnoses and onset of gambling symptoms as well as present and past treatments for gambling problems (e.g., pharmacotherapy, psychotherapy, counseling).

#### Yale-Brown Obsessive Compulsive Scale Adapted for Pathological Gambling (PG-YBOCS)

The PG-YBOCS (DeCaria et al. 1998) assesses gambling symptom severity on two subscales (thoughts/urges and behavior). The total score ranges from 0 to 40 points, distinguishing between subclinical (0–7), mild (8–15), moderate (16–23), severe (24–31), and extreme (32–40) severity. The PG-YBOCS shows a high reliability with high internal consistencies both for the total score and for the subscales (Cronbach’s α = 0.93–0.97; Pallanti et al. 2005). The current study used the German translation by Cremer and Hand (Cremer et al. 2001).

#### Gambling Attitudes and Beliefs Scale (GABS)

The GABS is a 35-item self-assessment questionnaire measuring dysfunctional attitudes and beliefs related to the onset and maintenance of gambling behavior (Breen and Zuckerman 1994). Items can be answered using a four-point scale (“fully applicable” to “not applicable”). The GABS shows good psychometric properties (Cronbach’s α = 0.89–0.93; Breen and Zuckerman 1999; Strong et al. 2004). We used the 15-item short version of the GABS developed by Strong et al. (2004). The total score of the GABS-15 ranges from 0 to 45 points.

#### Patient Satisfaction Questionnaire (German Acronym ZUF-8)

An adapted version of the ZUF-8 (Schmidt et al. 1989), the German equivalent of the Client Satisfaction Questionnaire (CEQ-8; Larsen et al. 1979), was used to evaluate subjective appraisal of the intervention. The questionnaire contains eight items that have to be answered using a four-point scale. The ZUF-8 shows good psychometric qualities with high internal consistency (Cronbach’s α = 0.87–0.93) and has good concurrent and prognostic validity (Schmidt and Wittmann 2002).

#### Subjective Appraisal of Modules Questionnaire

To evaluate the individual modules of the Gambling-MCT, we administered a ten-item questionnaire at the end of each session. Eight items (e.g., “The module was fun”) could be answered on a five-point rating scale (ranging from “does not apply at all” to “applies completely”), while two items used an open answer format (“I liked today: (...)”, “I did not like today: (...)”). Two items (“I would recommend the metacognitive training to a friend with similar problems” and “I want to come to the next training session”) were added to the questionnaire later. Therefore, not all participants provided data on these items.

#### Within-Session and Adverse Effects Questionnaire

To assess within-session changes, a questionnaire developed by our research group was administered before and after each module. It contained 15 items that had to be answered on a five-point scale (ranging from “does not apply at all” to “applies completely”) that assessed occurrence of and impairment by gambling thoughts, control over gambling, abstinence motivation (abstinence intentions [“I want to stop gambling”], abstinence confidence [“I think I can manage to stop gambling”], and efforts to resist gambling thoughts [“I am trying hard to resist gambling thoughts”]), emotional state (mood and restlessness), cognitive distortions (illusion of control and chasing), and positive associations with gambling as well as two module-specific items (e.g., module 8 on relapse prevention: “I know strategies to avoid a relapse”). Higher scores indicate higher impairment. Because the questionnaire was added to the study during the course of the trial, not all participants provided within-session ratings for all modules they attended.

### Intervention

We developed a metacognitive training for problem and pathological gambling (Gambling-MCT) in order to address and alleviate the severe distress experienced by problem and pathological gamblers. Gambling-MCT aims to bridge the treatment gap resulting from individual and structural treatment barriers. The training builds upon current empirical findings on (meta-)cognitive distortions associated with problem or pathological gambling behavior (for an overview, see the electronic supplementary material). As comorbid depressive symptoms often interact with gambling pathology, Gambling-MCT addresses gambling-specific as well as depressive cognitive biases. The training consists of eight modules, including four modules on (meta-)cognition (attributional style, probabilities I and II, memory), as well as modules on self-esteem and mood, debt regulation, urge to gamble, and relapse prevention (see Table1). A more detailed description of the modules is given in the supplementary material (supplementary material B). Metacognitive training for problem and pathological gamblers followed the design and concept of already established versions of metacognitive training, such as ones for psychosis (MCT; Moritz et al. 2014) and depression (D-MCT; Jelinek et al. 2015). Each module deals with an independent topic, which thus allows for an open group format and implementation in both outpatient and inpatient settings. Training materials are presented in a PowerPoint presentation, which enables a standardized implementation. To help consolidate training effects and to support transfer into everyday life, participants receive homework sheets, including a summary of the module’s content and further exercises. Due to its high level of standardization, besides psychologists and psychotherapists, social workers and addiction counselors can facilitate Gambling-MCT. This is deemed especially important for countries such as Germany, where the majority of outpatient treatment is provided by addiction counseling centers and over 90% of help-seeking individuals receive outpatient addiction counseling (Thaller et al. 2017). In addition, Gambling-MCT can be used as an additional treatment approach in outpatient psychotherapy practices or to bridge the waiting time for individual therapy. In inpatient settings, the intervention can be implemented as part of a multimodal treatment concept.

**Table 1:** The eight modules of Gambling-MCT

Module	Content
1: Attributional style	Different styles of (dysfunctional) attribution (e.g., self-serving bias)
	Attributional style for problem gambling and consequences: wins are attributed to personal skills, losses are attributed to chance or other circumstances
	Deducing more realistic attributions by using different sources of attributions (self, others, circumstances)
2: Probabilities I	Definition of cognitive distortions
	Gambling-related cognitive distortions focusing on one’s own impact on gambling outcome
	Near misses and gambler’s fallacy
	Exercises and information about probabilities in gambling
	Thought-action fusion
3: Self-esteem and mood	Definition and sources of self-esteem
	Identifying personal strengths
	Influence of mood and self-esteem on gambling behavior
	Tips to improve mood and self-esteem
4: Probabilities II	Definition of cognitive distortions
	Gambling-related cognitive distortions focusing on one’s own impact on gambling outcome
	Illusion of control and illusory correlations
	Superstitious thinking, rituals, and lucky charms
	Quiz on gambling myths
5: Memory	Memory capacity and false memories
	Memory biases in problem and pathological gambling: wins are more easily recalled than losses
	Mood congruency effect and the Pollyanna principle: negative mood evokes negative memories, positive mood evokes positive memories
	Cognitive distortion in depression: mental filter
6: Gambling urge	Triggers of gambling urge, positive and negative consequences of gambling behavior
	Functional analysis of gambling behavior: analyses of triggers and consequences (positive/negative) of gambling behavior in specific situations
	Triggers and acute urge to gamble
	Mindfulness meditation
7: Debt regulation	Downward spiral of debt and measures to stop it
	Upward spiral of money management: short-term and long-term measures to reduce debt
	Money-related dysfunctional cognitions and attitudes
8: Relapse prevention	Self-determined relapse prevention
	Personal triggers and warning signs of a relapse
	Emergency plan
	Depression and gambling

The modules include entertaining exercises that help participants to uncover and work on distorted thinking in a playful manner. In addition, numerous pictures, videos, and illustrations in the slides are meant to create a stimulating training atmosphere. Ideally, between three and ten patients participate in each session. In this study, the training was carried out in the two recruitment centers by trained psychologists (level of education: B.Sc. or higher) on a weekly basis. Each session lasted 60 min. We assume that the low-threshold approach, the open group format, and the stimulating training atmosphere of Gambling-MCT contribute to overcoming the treatment barriers faced by problem and pathological gamblers.

### Statistical Analyses

Symptom changes from baseline to post, subjective appraisals, and demographic and baseline characteristics were analyzed using SPSS^®^ version 23 (IBM Corp 2015). To calculate symptom changes over time (pre/post), paired sample *t*-tests (two-sided, α = 0.05) were conducted for the intention-to-treat sample (all participants), completers (completion of baseline and post assessments, participation in at least one module), and the per-protocol sample (completion of baseline and post assessments, participation in at least four modules). Dependent variables were PG-YBOCS total score, PG-YBOCS subscales (thoughts and behavior), and GABS total score at pre and post assessment. As no gold standard method exists for estimating missing data (Fan et al. 2020), two kinds of intention-to-treat analyses were conducted: multiple imputations (MI) and last observation carried forward (LOCF). Mean values, standard deviations, and response frequencies (percent) were calculated to evaluate patients’ subjective appraisals of the intervention.

The analysis that evaluated within-session changes was conducted using linear mixed-effect models (LMMs) by means of RStudio (RStudio Team 2019) as this method is recommended for analyzing clustered data (Hox 2010; Raudenbush and Bryk 2002). The strategy of data analysis was based on our research group’s previous publications (Miegel et al. 2019; Schneider et al. 2018). We used a two-level structure with time as the level 1 factor (repeated measures pre and post session) and subject as the level 2 factor. We analyzed within-session changes for each item and each module. We also calculated changes due to treatment over time using LMM (i.e., changes from first to last treatment session). For both analyses, we tested random slopes. Random slope and fixed slope models were compared using analysis of variance (ANOVA), and model fit was evaluated using the Akaike information criterion (AIC). The AIC is a commonly used criterion for model selection in LMM. The AIC considers two parameters: (1) the log likelihood (goodness of fit) and (2) the simplicity of the model. The lower the AIC, the better the model fit (Xu et al. 2015). The following R packages were used for data analyses: (1) lme4 package (Bates et al. 2015) was used for model computation and (2) lmerTest (Kuznetsova et al. 2017) was used for calculating *p*-values. Maximum likelihood estimation was used for parameter calculation (Nakagawa and Schielzeth 2013). Further information on the calculation of LMM in this study as well as specific equations can be found in the electronic supplementary material (supplementary material C).

## Results

Thirty-eight individuals with gambling problems were recruited over a period of one and a half years (June 2018 to December 2019), thirteen of whom had to be excluded for violating the inclusion criteria (*n* = 2) or declining to participate in the study (*n* = 11). The final sample consisted of 25 participants. Baseline demographics and psychopathology are displayed in Table 2. The vast majority of participants were male (92%, *n* = 23). Most participants (72%, *n* = 18) were recruited in the two treatment facilities (64% counseling center, *n* = 16; 8% outpatient institution for psychotherapy, *n* = 2). The rest (28%, *n* = 7) learned of the study through a Google AdWords campaign. Participants showed moderate severity of gambling symptoms (*M* = 16.48, *SD* = 8.53) and dysfunctional gambling attitudes (*M* = 20.04, *SD* = 6.41) as assessed with the PG-YBOCS and the GABS. On average, participants attended four of the eight sessions (*M* = 4.16, *SD* = 2.84). All eight modules were completed by 24% (*n* = 6) of the sample; 24% (*n* = 6) only visited one module. Almost three out of four (72%, *n* = 18) completed the post assessment. Completion rate (i.e., completion of baseline and post assessment; 72% vs. 71%) and module attendance (*t*(23) = 0.17, *p* = 0.865) did not differ between participants recruited at the two treatment centers, who were therefore already seeking help, and those, who were recruited via Google AdWords. Approximately one-quarter (24%, *n* = 6) of the sample was undergoing other treatment during the study period, including CBT (8%, *n* = 2), antidepressants (8%, *n* = 2), inpatient interval treatment (4%, *n* = 1), and psychiatrist consultation (4%, *n* = 1). Approximately one-third (36%, *n* = 9) had used mental health care in the past (e.g., psychotherapy, pharmacotherapy, counseling).

**Table 2:** Sociodemographic characteristics and psychopathology. Means and standard deviations (in brackets) or frequency (percent)

Variable		Sample (*N* = 25)
*Demographics*		
Gender	Female	2 (8%)
	Male	23 (92%)
Age (in years)		40.16 (12.72)
Highest educational level	No school-leaving qualification	2 (8%)
	Lower secondary school	2 (8%)
	Secondary school	12 (48%)
	Higher education entrance qualification	9 (28%)
Years of gambling		12.28 (11.25)
Currently employed		18 (72%)
Diagnosis	Pathological gambling	5 (20%)
	Depression	5 (20%)
	Substance-related disorder	1 (4%)
	Other	2 (8%)
Other treatment/therapy	Current	6 (24%)
	Past	9 (36%)
*Psychopathology*		
PG-YBOCS	Thoughts	9.28 (4.55)
	Behavior	7.20 (4.80)
	Total	16.48 (8.53)
GABS		20.04 (6.41)

PG-YBOCS = Yale-Brown Obsessive Compulsive Scale adapted for Pathological Gambling; GABS = Gambling Attitudes and Beliefs Scale

### Intention-to-Treat and Per-Protocol Analyses

Intention-to-treat analyses using both MI and LOCF showed significant symptom reductions on all measures with small to moderate effect sizes (*d*_*z*_ = 0.37—0.79; see Table 3), except for the behavior subscale of the PG-YBOCS. In the per-protocol analyses as well as analyses of the completer sample, significant symptom changes were observed on all scales, with moderate to strong effect sizes for completers (*d*_*z*_ = 0.47–1.02) and the PP sample (*d*_*z*_ = 0.73–1.37). A Mann–Whitney U Test was calculated in the PP sample to detect differences in symptom reduction among participants who were undergoing other treatment during the study period, and participants who were being treated with Gambling-MCT only. No significant differences emerged on any outcome measure (GABS: *U* = 7.50, *Z* =  − 1.78, *p* = 0.076; PG-YBOCS total: *U* = 7.50, *Z* =  − 1.78, *p* = 0.076; PG-YBOCS thoughts: *U* = 8.50, *Z* =  − 1.67, *p* = 0.100; PG-YBOCS behavior: *U* = 10.50, *Z* =  − 1.45, *p* = 0.164).

**Table 3:** Symptom change over time (intention-to-treat and per-protocol analyses)

Variable	Mean (*SD*)		Paired *t*-test (two-tailed)		
	Baseline	Post	Completers (*n* = 18)	Per-protocol (*n* = 12)	Intention-to-treat analyses: MI/LOCF (*N* = 25)
PG Y-BOCS total	15.50 (8.64)	8.78 (7.73)	*t*(17) = 3.10, *p* = .007, *d*_*z*_ = 0.73	*t*(11) = 3.40, *p* = .006, *d*_*z*_ = 0.98	*t*(24) = 2.36, *p* = .022, *d*_*z*_ = 0.47
					*t*(24) = 2.90, *p* = .008, *d*_*z*_ = 0.58
PG Y-BOCS thoughts	8.50 (4.41)	5.33 (3.68)	*t*(17) = 2.63, *p* = .018, *d*_*z*_ = 0.47	*t*(11) = 2.52, *p* = .029, *d*_*z*_ = 0.73	*t*(24) = 2.48, *p* = .015, *d*_*z*_ = 0.50
					*t*(24) = 2.51, *p* = .019, *d*_*z*_ = 0.50
PG Y-BOCS behavior	7.00 (4.96)	3.44 (4.30)	*t*(17) = 3.08, *p* = .007, *d*_*z*_ = 0.62	*t*(11) = 3.70, *p* = .003, *d*_*z*_ = 1.07	*t*(24) = 1.83, *p* = .072, *d*_*z*_ = 0.37
					*t*(24) = 2.89, *p* = .008, *d*_*z*_ = 0.58
GABS	19.67 (5.65)	10.06 (8.39)	*t*(17) = 4.32, *p* < .001, *d*_*z*_ = 1.02	*t*(11) = 4.73, *p* = .001, *d*_*z*_ = 1.37	*t*(24) = 2.96, *p* = .004, *d*_*z*_ = 0.59
					*t*(24) = 3.94, *p* = .001, *d*_*z*_ = 0.79

Means and standard deviations (*SD*) were calculated for study completers (pairwise data); MI = multiple imputation; LOCF = last observation carried forward; PG-YBOCS = Yale-Brown Obsessive Compulsive Scale adapted for Pathological Gambling; GABS = Gambling Attitudes and Beliefs Scale

### Linear Mixed-Effect Models

The AICs for all tested models of within-session analysis and analysis over the duration of the treatment are displayed in the supplementary material (supplementary material D). Calculation of treatment effects over the intervention period using LMM (i.e., changes from first to last session in within-session items) showed best model fit of random intercept models for all items except abstinence confidence, financial trigger, illusion of control, and chasing. Significant reductions in gambling thoughts (*b* =  − 0.08, 95% CI [− 0.15, − 0.01], *p* = 0.017), control over gambling (*b* =  − 0.12, 95% CI [− 0.21, − 0.04], *p* = 0.005), impairment due to gambling thoughts (*b* =  − 0.10, 95% CI [− 0.17, − 0.03], *p* = 0.005), financial trigger (*b* =  − 0.14, 95% CI [− 0.23, − 0.04], *p* = 0.005), sadness (*b* =  − 0.14, 95% CI [− 0.21, − 0.06], *p* < 0.001), restlessness (*b* =  − 0.14, 95% CI [− 0.22, − 0.06], *p* = 0.001), positive associations with gambling (*b* =  − 0.08, 95% CI [− 0.13, − 0.02], *p* = 0.005), illusion of control (*b* =  − 0.10, 95% CI [− 0.17, − 0.02], *p* = 0.015), and chasing (*b* =  − 0.07, 95% CI [− 0.13, − 0.02], *p* = 0.011) were found. Items related to abstinence motivation (abstinence intentions, abstinence confidence, and efforts to resist gambling thoughts) remained unchanged during the study period.

The within-session changes are displayed in Table 4. LMM analysis of within-session changes showed that for the majority of analyses the model that provided the best fit was the random intercept model. In contrast, for module 2 (probabilities I), random slope models demonstrated the best fit for seven of the twelve constructs assessed (gambling thoughts, effort to resist gambling thoughts, abstinence confidence, restlessness, positive associations, illusion of control, chasing). Random slope models for impairment due to gambling thoughts, abstinence intention, financial trigger, positive associations, and illusion of control were found to best fit module 1 (attributional style). Moreover, within-session changes in abstinence confidence were best described by random slope models for module 3 (self-worth and mood), module 4 (probabilities II), and module 8 (relapse prevention). The superiority of the fit of the random slope model for these analyses points out a significant variance in participants’ level of these constructs at the beginning of the sessions as well as in their within-session change. After Bonferroni correction for multiple testing, no significant within-session changes remained. All uncontrolled changes have to be interpreted with caution. Calculations showed adverse effects for the second module (probabilities I) on occurrence of gambling thoughts, perceived control over gambling, positive associations with gambling, and financial trigger (“If I had more money, I would start gambling again”). After module 1 (attributional style), participants reported higher impairment due to gambling thoughts compared to all other modules, but at the same time fewer gambling thoughts were reported. After module 8 (relapse prevention), stronger efforts to resist gambling were reported. Module 3 (self-esteem and mood) led to a reduction in reported sadness of participants.

**Table 4:** Results of linear mixed-effects models within-session analyses presented as beta values for fixed effect (B) before Bonferroni correction

Module	Gambling thoughts		Control over gambling		Efforts to resist thoughts		Impairment due to gambling thoughts		Abstinence intention		Abstinence confidence	
Range (intercept)	1.04–1.37, *p* < .001		1.33–1.45, *p* < .001		1.48–1.66, *p* < .001		0.92–1.21, *p* ≤ .001		0.92–0.97, *p* < .001		0.41–0.50, *p* ≤ .001–.003	
	*B*	*p*	*B*	*p*	*B*	*p*	*B*	*p*	*B*	*p*	*B*	*p*
Module 1	− 0.65	**.007**	− 0.16	.600	0.05	.848	0.71	.**027**	0.00	.967	0.05	.691
Module 2	0.96	**.008**	0.66	**.014**	0.60	.067	− 0.13	.577	− 0.02	.675	− 0.01	.921
Module 3	− 0.19	.438	0.47	.105	− 0.16	.519	− 0.17	.528	0.05	.470	0.09	.503
Module 4	0.04	.885	− 0.28	.404	0.01	.963	− 0.09	.738	− 0.03	.617	0.09	.578
Module 5	− 0.33	.244	− 0.03	.923	0.22	.471	− 0.32	.295	− 0.02	.747	− 0.12	.382
Module 6	0.09	.751	− 0.18	.572	− 0.08	.774	0.09	.738	− 0.03	.629	− 0.03	.789
Module 7	0.01	.959	− 0.42	.209	− 0.16	.576	− 0.11	.697	0.03	.645	− 0.08	.548
Module 8	− 0.09	.742	− 0.38	.243	− 0.61	.**025**	− 0.27	.346	− 0.02	.758	0.00	.988
Random parts												
Time	0.23–0.62		0.86–0.95		0.45–0.70		0.44–0.71		0.04		0.12–0.14	
Subject	0.27–0.45		0.20–0.29		0.34–0.39		0.16–0.44		0.11–0.12		0.06–0.13	
ICC	.40–.73		.18–.25		.33–.54		.18–.51		.75–.78		.00–.46	
NID	21		20		21		20		21		20	
Observations (*N*)	91		92		94		92		94		93	

Module	Financial trigger		Sadness		Restlessness		Positive associations		Illusion of control		Chasing	
Range (intercept)	0.94–1.22, *p* < .001		0.61–0.69, *p* = .001–.003		0.75–0.81, *p* < .001		1.28–1.56, *p* < .001		0.88–1.15, *p* < .001		0.90–1.05, *p* < .001	
	*B*	*p*	*B*	*p*	*B*	*p*	*B*	*p*	*B*	*p*	*B*	*p*
Module 1	− 0.49	.204	0.17	.419	− 0.14	.565	− 0.01	.963	− 0.08	.735	0.07	.618
Module 2	0.47	.**025**	0.00	.987	− 0.02	.940	0.46	**.025**	0.39	.097	0.32	.076
Module 3	− 0.07	.757	− 0.41	**.043**	0.17	.473	− 0.20	.173	− 0.29	.126	− 0.19	.150
Module 4	0.08	.756	− 0.17	.449	− 0.53	.057	− 0.28	.100	− 0.10	.546	0.10	.493
Module 5	0.07	.790	0.31	.197	0.22	.450	0.00	.987	− 0.03	.850	0.03	.866
Module 6	− 0.15	.536	− 0.05	.815	− 0.05	.862	− 0.18	.240	− 0.02	.887	− 0.10	.496
Module 7	0.37	.174	0.17	.468	0.08	.764	− 0.01	.943	− 0.02	.896	− 0.08	.597
Module 8	− 0.24	.342	0.10	.677	0.10	.720	0.04	− 0.02	.896	− 0.08	.597	.186
Random parts												
Time	0.41–0.59		0.44–0.46		0.47–0.65		0.15–0.23		0.08–0.22		0.11–0.19	
Subject	0.31–0.63		0.12		0.06–0.26		0.43–0.86		0.11–0.71		0.31–0.35	
ICC	.34–.59		.21		.08–.39		.68–.86		.50–.82		.62–.78	
NID	20		20		20		20		20		20	
Observations (*N*)	93		93		91		93		93		93	

ICC = intraclass correlation; NID = normally and independently distributed; modules: 1 = attributional style, 2 = probabilities I, 3 = self-worth and mood, 4 = probabilities II, 5 = memory, 6 = gambling urge, 7 = debt regulation, 8 = relapse prevention; significant uncorrected effects in bold (α < .05)

Two items were not included in the LMM analyses as they were assessed for those modules only. Ratings of those items will be used for adaption of Gambling-MCT but are not further discussed in this article. Another item had to be excluded from the analyses because of ambiguous interpretation by participants (“If I stopped gambling, all my problems would be solved”). Some participants referred to their gambling problems only when answering the item, while others referred to all their personal problems, including those that could not be changed by their behavior (e.g., death of a relative, physical health problems).

### Subjective Appraisal

The subjective appraisal of Gambling-MCT was good (see Table 5). Out of the 18 participants who completed the post assessment, more than 90% (*n* = 17) rated the quality of the intervention as good to excellent. Almost 90% (*n* = 16) were satisfied with the program and would recommend it to individuals with similar problems. More than 80% (*n* = 15) stated that Gambling-MCT helped them cope with their problems more successfully and that they would participate again. The intervention met the needs and expectations of almost 80% (*n* = 14) of participants.

**Table 5:** Subjective appraisal of metacognitive training for problem gamblers (adapted from the Patient Satisfaction Questionnaire; ZUF-8). Means and standard deviations (in brackets) and positive appraisal (in percent) for the completer sample

Item	Completer (*n* = 18)	
	Mean (SD)	Positive appraisal
How do you rate the quality of the metacognitive training? (excellent, good vs. okay, not good)	3.22 (0.55)	94.4%
Did you receive the type of treatment you expected to receive? (absolutely, a lot vs. a little, not at all)	3.00 (0.84)	77.8%
To what extent did the metacognitive training meet your needs? (absolutely, a lot vs. a little, not at all)	3.11 (0.90)	77.8%
Would you recommend the metacognitive training to a friend with similar symptoms? (yes, probably yes vs. probably not, no)	3.56 (0.70)	88.9%
How satisfied are you with the extent of help you have received through using the metacognitive training? (very satisfied, mostly satisfied vs. somewhat satisfied, dissatisfied)	3.11 (1.02)	77.7%
Did the metacognitive training help you to cope with your problems more successfully? (absolutely, a lot vs. a little, not at all)	3.56 (0.78)	83.3%
How satisfied are you with the metacognitive training in general? (very satisfied, mostly satisfied vs. somewhat satisfied, dissatisfied)	3.39 (0.85)	88.9%
Would you use the metacognitive training again? (yes, probably yes vs. probably not, no)	3.44 (0.78)	83.3%

Scores range from 1 to 4. Higher scores indicate higher satisfaction with treatment

Subjective appraisal of individual modules can be derived from Table 6 that gives percentages and absolute numbers of participants taking part in each module. Over 90% of participants confirmed that seven of the eight modules were fun. The session on self-esteem and mood was rated as fun by slightly less than two-thirds of the participants (64%). The majority of participants reported that they learned something new in all modules, and all participants affirmed this for modules 5 (memory) and 8 (relapse prevention). Moreover, all participants reported they intended to adopt what they had learned in the modules about targeting gambling urge, debt regulation, and relapse prevention in their daily routine. Depending on the module, 80% to 100% rated the content of the session personally relevant and understandable. After all the modules, at least 90% expressed their intention to come to the next training session.

**Table 6:** Subjective appraisal of each module. Means and standard deviations (in brackets) and positive appraisal* (in percent)

Item	Module 1 (*n* = 15)	Module 2 (*n* = 19)	Module 3 (*n* = 14)	Module 4 (*n* = 11)	Module 5 (*n* = 10)	Module 6 (*n* = 13)	Module 7 (*n* = 11)	Module 8 (*n* = 11)
The module was fun	4.53 (0.52) 100%	4.42 (0.61) 94.8%	4.00 (1.04) 64.3%	4.63 (0.50) 100%	4.40 (0.70) 90.0%	4.77 (0.60) 92.3%	4.64 (0.50) 100%	4.72 (0.47) 100%
I learned something new in the module	3.93 (0.70) 86.6%	3.84 (1.17) 73.7%	4.00 (0.78) 71.5%	3.91 (1.14) 72.8%	4.40 (0.52) 100%	4.61 (0.87) 93.3%	4.45 (0.82) 81.8%	4.55 (0.52) 100%
I want to use what I learned in the module in my daily routine	4.40 (0.63) 93.4%	4.33 (0.69) 88.8%^b^	4.00 (0.68) 78.5%	4.45 (0.69) 90.9%	4.30 (0.67) 90.0%	4.46 (0.52) 100%	4.64 (0.50) 100%	4.60 (0.52) 100%
The metacognitive training helps me to cope with my gambling problems	4.27 (0.70) 86.7%	4.06 (0.56) 88.6%^c^	3.86 (0.86) 71.4%	4.18 (0.75) 81.9%	4.30 (0.67) 90.0%	4.54 (0.52) 100%	4.64 (0.50) 100%	4.64 (0.67) 90.9%
The content of the module is personally relevant for me	4.13 (0.74) 93.4%	4.17 (0.92) 88.9%^b^	4.07 (1.07) 85.7%	4.45 (0.69) 90.9%	4.20 (0.42) 100%	4.38 (1.33) 84.6%	4.73 (0.65) 90.9%	4.64 (0.67) 90.9%
I (partially) did not understand the content of the module.**	1.80 (1.21) 80.0%	1.11 (0.33) 100%^d^	1.75 (1.36) 100%^e^	1.18 (0.40) 100%	1.50 (1.27) 90.0%	1.15 (0.55) 92.3%	1.18 (0.60) 100%	1.00 (0.00) 100%
I would recommend the metacognitive training to a friend with similar problems	4.53 (0.52) 100%^a^	4.28 (0.67) 88.9%^b^	3.85 (1.07) 69.3%^f^	4.30 (0.95) 90.0%^g^	4.33 (1.12) 77.8%^d^	4.64 (0.50) 100%^h^	4.80 (0.42) 100%^j^	4.60 (0.48) 100%^i^
I want to come to the next training session	4.92 (0.28) 100%^a^	4.89 (0.32) 100%^b^	4.85 (0.38) 100%^f^	4.90 (0.32) 100%^g^	4.89 (0.33) 100%^d^	5.00 (0.00) 100%^h^	5.00 (0.00) 100%^j^	4.70 (0.67) 90.0%^i^

Coding: 1 (does not apply at all) to 5 (totally applies); *positive appraisal = totally applies, somewhat applies **positive appraisal = somewhat does not apply, does not apply at all; ^a^ *n* = 13; ^b^ *n* = 18; ^c^ *n* = 17; ^d^ *n* = 9; ^e^ *n* = 12; ^f^ *n* = 13; ^g^ *n* = 10; ^h^ *n* = 11, ^i^ *n* = 10; modules: 1 = attributional style, 2 = probabilities I, 3 = self-worth and mood, 4 = probabilities II, 5 = memory, 6 = gambling urge, 7 = debt regulation, 8 = relapse prevention

## Discussion

Problem and pathological gambling results in severe impairments in psychosocial functioning and a low quality of life (Cowlishaw et al. 2016; Weinstock et al. 2017). At the same time, only a small percentage of those affected are in treatment (Meyer et al. 2011), which reflects both a lack of available treatment programs (Thaller et al. 2017) and treatment barriers on the patient side (Suurvali et al. 2009). In order to narrow this treatment gap, we developed a low-threshold metacognitive group training for individuals with gambling problems (Gambling-MCT).

### Main Findings

Significant symptom reduction in gambling-related cognitive distortions (moderate to large effect sizes), as measured with the GABS, emerged from baseline to post assessment. Moreover, overall symptom severity, as measured with the PG-YBOCS, declined at small to large effect sizes. Speaking for the safety of the approach, linear mixed models evaluating session-specific changes did not show any adverse effects in symptomatology after Bonferroni correction for multiple testing. In fact, analysis of changes over the duration of the treatment indicated symptom reduction over the treatment course, as measured with the Within-Session and Adverse Effects questionnaire. However, deterioration on some items (occurrence of gambling thoughts, perceived control over gambling, positive associations with gambling, financial trigger) were observed after module 2 (probabilities I) compared to all other modules. Although none of these adverse effects remained significant after Bonferroni correction, we intend to revise this module. Compared with the other modules of Gambling-MCT, this module contained the most gambling-related pictures, which may have triggered the gambling urge (Ashrafioun et al. 2012).

Gambling-MCT demonstrated high acceptance among problem and pathological gamblers in this trial. Satisfaction with treatment was comparable to the level of acceptance in other treatment studies targeting problem and pathological gamblers undergoing dialectical behavioral group therapy (Christensen et al. 2013), couples therapy (Nilsson et al. 2018), and internet-based interventions (Bücker et al. 2018; Casey et al. 2017; Jonas et al. 2019) and was better than the appraisal of e-mail counseling (Jonas et al. 2019) as well as a monitoring, feedback, and support program (Casey et al. 2017). This high level of treatment satisfaction along with the absence of negative side effects and good adherence (completion rate: 72%) support the feasibility of Gambling-MCT.

The completion rate of baseline and post assessments (72%) was good; completion rates in other treatment studies range from 38% (Ladouceur et al. 2015) to 100% (Toneatto 2016). On average, participants attended half of the training sessions. Approximately one-quarter finished all modules; another quarter attended only one module. These findings corroborate the clinical observation that motivation for treatment in problem and pathological gamblers fluctuates, resulting in high dropout rates and postponements or cancellations of appointments as well as discontinuation of therapy (Ronzitti et al. 2017; Toneatto 2005). To further improve module attendance, Gambling-MCT may be extended through motivational techniques such as motivational interviewing (Miller and Rollnick 2002). Such techniques may increase treatment motivation in problem and pathological gamblers (Jonsson et al. 2020; Pfund et al. 2020).

### Treatment Gap for Problem and Pathological Gamblers

We hypothesized that the low-threshold and normalizing treatment approach, the cost-effectiveness, the open group format, and the stimulating training atmosphere of Gambling-MCT would contribute to reducing the treatment gap for problem and pathological gamblers by addressing both treatment barriers of the health system (e.g., lack of treatment programs, financial concerns) as well as barriers on the patient side (e.g., fear of failing in therapy, shame). Despite great effort and the implementation of different recruitment methods (i.e., media advertisements and recruitment through treatment facilities) that have been shown to recruit individuals with varying gambling severity (i.e., problem and pathological gamblers; Williams et al. 2010), only 25 participants were recruited over a period of almost one and a half years. This is in line with prior observations that recruitment of participants is a major challenge in gambling research (Toneatto 2005) due to the barriers experienced by problem and pathological gamblers (see introduction). The problems in recruitment of participants may suggest, contrary to our hypothesis, that the implementation of low-threshold metacognitive training did not fully overcome the treatment barriers of problem and pathological gamblers. This indicates that barriers on the patient side seem to be more decisive than the dearth of treatment options. Patient-related barriers such as problem denial may be especially hard to overcome (Gainsbury et al., 2014; Khayyat-Abuaita et al. 2015). However, it may also be possible that certain features of Gambling-MCT were not sufficiently highlighted in the study advertising. Future studies on Gambling-MCT should address this shortcoming by pointing out the advantages of the group training in the study advertising (e.g., video clips showing examples of the program and feedback from participants) because positive treatment perceptions have been associated with higher treatment initiation in the past (Khayyat-Abuaita et al. 2015).

To conclude, addressing treatment barriers as well as expectations and wishes of patients regarding treatment, which we are only beginning to understand, is important in reducing the treatment gap for individuals with gambling problems. Previous studies on treatment barriers show several methodological weaknesses, namely, small sample sizes, retrospective assessment via self-report, and a lack of assessment of important sociodemographic variables (Suurvali et al. 2009). Future studies need to address these shortcomings. Subsequently, based on these findings, treatment programs such as Gambling-MCT need to be adapted to better address fears and reservations about treatment initiation and to meet the needs and wishes for intervention held by problem and pathological gamblers.

### Limitations

We would like to acknowledge some limitations of the present study. First, while the results tentatively demonstrate the safety and feasibility of the intervention, due to the uncontrolled design of the pilot study, the results on the efficacy of Gambling-MCT are not fully reliable. We cannot rule out that symptom changes might be due to reasons other than participation in Gambling-MCT, namely, the effects of concurrent participation in other treatment programs or spontaneous remission since approximately one-third of all gamblers manages to quit gambling without seeking help (Cunningham et al. 2009; Meyer et al. 2011; Slutske 2006). The first is unlikely as no significant differences emerged between participants using versus not using other treatment programs. Also, the latter might not be as common in this study as it is most likely to occur in patients with low symptom severity (Meyer et al. 2011); participants in the Gambling-MCT in this study on average showed moderate gambling severity at baseline. Nevertheless, effects of spontaneous remission are possible.

Second, we did not differentiate between diagnosed and non-diagnosed pathological gamblers but included all participants with subjective gambling problems because we consider the need for treatment among problem gamblers to be high (see introduction). This liberal inclusion strategy may lead to a higher chance of a Type I error. Third, due to the difficulties in recruitment discussed above, the sample size of the study was rather small, which limited the generalizability of the results. Moreover, not all participants provided data on two items of the module-specific subjective appraisal questionnaire and within-session ratings for all modules they attended as these two measures were added after the study period had started. In addition, participants only attended, on average, four of the eight modules. Due to the small sample size, these two points limit the results. Future studies need to evaluate Gambling-MCT with larger samples. Fourth, data were collected using self-report measures. Although these are time efficient, they are susceptible to biases such as social desirability or tendencies toward extreme response behavior. To address these biases, anonymous assessments and instruments with high psychometric qualities (GABS, PG-YBOCS) were used. Nevertheless, external ratings should be included in future studies. Finally, as Gambling-MCT addresses gambling-related and depressive symptoms, future studies should include measures assessing depressive symptomatology. Depressive symptoms were not assessed in the current trial. Accordingly, it cannot be discerned whether the reduction of gambling-related cognitive distortions ans gambling symptoms was caused by a decline in depressive symptoms or gambling-specific cognitive distortions or both. Furthermore, it is not yet known whether addressing depressive cognitive distortions in Gambling-MCT is necessary to yield an effect.

All in all, to assess valid data on the efficacy and acceptance of Gambling-MCT, randomized controlled trials with large sample sizes and follow-up assessments are needed. We did not conduct such a trial as the primary aim of this pilot study was to evaluate the feasibility, acceptance and safety of our approach as a first step. We plan to conduct a randomized controlled trial to evaluate long- and short-term effectiveness of the intervention.

### Implications

This trial offers first evidence of the acceptance and safety of Gambling-MCT, which supports the feasibility of the training. However, participants’ attendance of the modules in this trial was not fully satisfactory. Moreover, recruitment of participants remains a critical task in gambling research. Future studies need to evaluate the effectiveness of the intervention in randomized controlled trials with a larger sample to provide data on short- and long-term effects of the intervention. Reasons for non-initiation of treatment need to be further investigated so that treatment programs and recruitment strategies can be adapted to the needs and reservations of individuals with gambling problems. Moreover, including motivational strategies in study advertising, such as highlighting reasons to initiate treatment and creating positive treatment perceptions, as well as offering treatment programs that contain strategies from MI, may raise treatment motivation.

## Electronic Supplementary Material

Below is the link to the electronic supplementary material.

Supplementary file1 (DOC 715 kb)

## References

[CR1] American Psychiatric Association (2013). Diagnostic and statistical manual of mental disorders.

[CR2] Ashrafioun L, McCarthy A, Rosenberg H (2012). Assessing the impact of cue exposure on craving to gamble in university students. Journal of Gambling Studies.

[CR84] Bates D, Mächler M, Bolker B, Walker S (2015). Fitting linear mixed-effects models using lme4. Journal of Statistical Software.

[CR3] Bilevicius E, Single A, Bristow LA, Foot M, Ellery M, Keough MT, Johnson EA (2018). Shame mediates the relationship between depression and addictive behaviours. Addictive Behaviors.

[CR4] Blaszczynski A, Nower L (2002). A pathways model of problem and pathological gambling. Addiction.

[CR5] Breen RB, Zuckerman M (1994). The gambling beliefs and attitudes survey.

[CR6] Breen RB, Zuckerman M (1999). “Chasing” in gambling behavior: Personality and cognitive determinants. Personality and Individual Differences.

[CR7] Bücker L, Bierbrodt J, Hand I, Wittekind CE, Moritz S (2018). Effects of a depression-focused internet intervention in slot machine gamblers: A randomized controlled trial. PLoS ONE.

[CR8] Casey LM, Oei TPS, Raylu N, Horrigan K, Day J, Ireland M, Clough BA (2017). Internet-based delivery of cognitive behaviour therapy compared to monitoring, feedback and support for problem gambling: A randomised controlled trial. Journal of Gambling Studies.

[CR9] Christensen DR, Dowling NA, Jackson AC, Brown M, Russo J, Francis KL, Umemoto A (2013). A proof of concept for using brief dialectical behavior therapy as a treatment for problem gambling. Behaviour Change.

[CR10] Ciccarelli M, Griffiths MD, Nigro G, Cosenza M (2017). Decision making, cognitive distortions and emotional distress: A comparison between pathological gamblers and healthy controls. Journal of Behavior Therapy and Experimental Psychiatry.

[CR11] Clark L (2010). Decision-making during gambling: An integration of cognitive and psychobiological approaches. Philosophical Transactions of the Royal Society B: Biological Sciences.

[CR12] Clark L, Lawrence AJ, Astley-Jones F, Gray N (2009). Gambling near-misses enhance motivation to gamble and recruit win-related brain circuitry. Neuron.

[CR13] Clarke D, Abbott M, DeSouza R, Bellringer M (2007). An overview of help seeking by problem gamblers and their families including barriers to and relevance of services. International Journal of Mental Health and Addiction.

[CR14] Cocker PJ, Winstanley CA (2015). Irrational beliefs, biases and gambling: Exploring the role of animal models in elucidating vulnerabilities for the development of pathological gambling. Behavioural Brain Research.

[CR15] Cowlishaw S, Hakes JK, Dowling NA (2016). Gambling problems in treatment for affective disorders: Results from the National Epidemiologic Survey on Alcohol and Related Conditions (NESARC). Journal of Affective Disorders.

[CR16] Cowlishaw S, Merkouris SS, Dowling NA, Anderson C, Jackson A, Thomas SA (2012). Psychological therapies for pathological and problem gambling. Cochrane Database of Systematic Reviews.

[CR17] Cremer, J., Zorawski, M., & Hand, I. (2001). *South Oaks Gambling Screen (SOGS), überarbeitete Fassung von H. R. Lesieur und S. B. Blume, 1993 und Pathological Gambling-Modification of the Yale-Brown Obsessive-Compulsive Scale (PG-YBOCS) von C. DeCaria und E. Hollander, 1998 Autorisierte deutsche Übe*. https://spielerprojekt-prof-hand.vt-falkenried.de/images/Publikationen/Spieler/SOGSuPGYBOCS.pdf.

[CR18] Cunningham J, Hodgins DC, Toneatto T (2009). Natural history of gambling problems: Results from a general population survey. Sucht: Zeitschrift für Wissenschaft und Praxis.

[CR19] Dąbrowska K, Moskalewicz J, Wieczorek Ł (2017). Barriers in access to the treatment for people with gambling disorders. Are they different from those experienced by people with alcohol and/or drug dependence?. Journal of Gambling Studies.

[CR20] DeCaria, C. M., Hollander, E., Begaz, T., Schemeidler, J., Wong, C. M., Cartwright, C., & Mosovich, S. (1998). *Reliability and validity of a pathological gambling modification of the Yale-Brown Obsessive Compulsive Scale (PG-YBOCS): preliminary findings. Paper Presentation at the 12th National Conference on Problem Gambling*. 17–20.

[CR21] Dowling NA, Cowlishaw S, Jackson AC, Merkouris SS, Francis KL, Christensen DR (2015). Prevalence of psychiatric co-morbidity in treatment-seeking problem gamblers: A systematic review and meta-analysis. Australian and New Zealand Journal of Psychiatry.

[CR22] Dowling NA, Merkouris SS, Greenwood CJ, Oldenhof E, Toumbourou JW, Youssef GJ (2017). Early risk and protective factors for problem gambling: A systematic review and meta-analysis of longitudinal studies. Clinical Psychology Review.

[CR23] Eichner C, Berna F (2016). Acceptance and efficacy of metacognitive training (MCT) on positive symptoms and delusions in patients with schizophrenia: A meta-analysis taking into account important moderators. Schizophrenia Bulletin.

[CR24] Fan C, Wei L, Koch GG (2020). Methods for missing data handling in phase III clinical trials with nonnormal endpoints and nonnormal covariates. Statistics in Biopharmaceutical Research.

[CR25] Flavell JH (1979). Metacognition and cognitive monitoring: A new area of cognitive-developmental inquiry. American Psychologist.

[CR26] Fortune EE, Goodie AS (2012). Cognitive distortions as a component and treatment focus of pathological gambling: A review. Psychology of Addictive Behaviors.

[CR27] Gainsbury S, Hing N, Suhonen N (2014). Professional help-seeking for gambling problems: Awareness, barriers and motivators for treatment. Journal of Gambling Studies.

[CR28] Ginley MK, Rash CJ, Petry NM, Heinz A, Romanczuk-Seiferth N, Potenza MN (2019). Psychological interventions in gambling disorder. Gambling disorder.

[CR29] Gooding P, Tarrier N (2009). A systematic review and meta-analysis of cognitive-behavioural interventions to reduce problem gambling: Hedging our bets?. Behaviour Research and Therapy.

[CR30] Hodgins DC, Stea JN, Grant JE (2011). Gambling disorders. The Lancet.

[CR31] Hox JJ (2010). Multilevel analysis: Techniques and applications.

[CR32] Hunt CJ, Blaszczynski A, Heinz A, Romanczuk-Seiferth N, Potenza MN (2019). Gambling disorder as a clinical phenomenon. Gambling disorder.

[CR33] IBM Corp. (2015). *IBM SPSS statistics for Windows, Version 23.0*. Author.

[CR34] Jelinek, L., Hauschildt, M., & Moritz, S. (2015). *Metakognitives Training bei Depression*. Beltz

[CR35] Jonas B, Leuschner F, Eiling A, Schoelen C, Soellner R, Tossmann P (2019). Web-based intervention and email-counseling for problem gamblers: Results of a randomized controlled trial. Journal of Gambling Studies.

[CR36] Jonsson J, Hodgins DC, Munck I (2020). Reaching out to big losers leads to sustained reductions in gambling over 1 year: A randomized controlled trial of brief motivational contact. Addiction.

[CR37] Keough MT, Penniston TL, Vilhena-Churchill N, Bagby RM, Quilty LC (2018). Depression symptoms and reasons for gambling sequentially mediate the associations between insecure attachment styles and problem gambling. Addictive Behaviors.

[CR38] Kessler RC, Hwang I, LaBrie R, Petukhova M, Sampson NA, Winters KC, Shaffer HJ (2008). The prevalence and correlates of DSM-IV pathological gambling in the national comorbidity survey replication. Psychological Medicine.

[CR39] Khantzian EJ (1997). The self-medication hypothesis of substance use disorders: A reconsideration and recent applications. Harvard Review of Psychiatry.

[CR40] Khayyat-Abuaita U, Ostojic D, Wiedemann A, Arfken CL, Ledgerwood DM (2015). Barriers to and Reasons for Treatment Initiation among Gambling Help-line Callers. Journal of Nervous and Mental Disease.

[CR41] Kuznetsova A, Brockhoff PB, Christensen RHB (2017). Lmertest package: Tests in linear mixed effects models. Journal of Statistical Software.

[CR42] Ladouceur R, Fournier P-M, Lafond S, Boudreault C, Goulet A, Sévigny S, Simoneau H, Giroux I (2015). Impacts of a self-help treatment program for problem gamblers. The Canadian Journal of Addiction.

[CR43] Ladouceur R, Sévigny S (2005). Structural characteristics of video lotteries: Effects of a stopping device on illusion of control and gambling persistence. Journal of Gambling Studies.

[CR44] Larsen DL, Attkisson CC, Hargreaves WA, Nguyen TD (1979). Assessment of the client/patient satisfaction: Development of a general scale. Evaluation and Program Planning.

[CR45] Liu Y-C, Tang C-C, Hung T-T, Tsai P-C, Lin M-F (2018). The efficacy of metacognitive training for delusions in patients with schizophrenia: A meta-analysis of randomized controlled trials informs evidence-based practice. Worldviews of Evidence-Based Nursing.

[CR46] Lorains FK, Cowlishaw S, Thomas SA (2011). Prevalence of comorbid disorders in problem and pathological gambling: Systematic prevalence of comorbid disorders in problem and pathological gambling: Systematic review and meta-analysis of population surveys review and meta-analysis of population survey. Addiction.

[CR47] Melville KM, Casey LM, Kavanagh DJ (2007). Psychological treatment dropout among pathological gamblers. Clinical Psychology Review.

[CR48] Meyer, C., Rumpf, H. J., Kreuzer, A., de Brito, S., Glorius, S., Jeske, C., Kastirke, N., Porz, S., Schön, D., Westram, A., Klinger, D., Goeze, C., Bischof, G., & John, U. (2011). *Pathologisches Glücksspielen und Epidemiologie (PAGE): Entstehung, Komorbidität, Remission und Behandlung. Endbericht an das Hessische Ministerium des Inneren und für Sport*. Universitätsmedizin Greifswald, Institut für Epidemiologie und Sozialmedizin & Universität zu Lübeck, Forschungsgruppe S:TEP, Klinik für Psychiatrie und Psychotherapie.

[CR49] Miegel F, Cludius B, Hottenrott B, Demiralay C, Sure A, Jelinek L (2019). Session-specific effects of the Metacognitive Training for Obsessive-Compulsive Disorder (MCT-OCD). Psychotherapy Research.

[CR50] Miller WR, Rollnick S (2002). Motivational Interviewing: Preparing people for change (2. Aufl.).

[CR51] Moritz S, Andreou C, Schneider BC, Wittekind CE, Menon M, Balzan RP, Woodward TS (2014). Sowing the seeds of doubt: A narrative review on metacognitive training in schizophrenia. Clinical Psychology Review.

[CR52] Moritz S, Lysaker PH (2018). Metacognition—What did James H. Flavell really say and the implications for the conceptualization and design of metacognitive interventions. Schizophrenia Research.

[CR53] Nakagawa S, Schielzeth H (2013). A general and simple method for obtaining R2 from generalized linear mixed-effects models. Methods in Ecology and Evolution.

[CR54] Nilsson A, Magnusson K, Carlbring P, Andersson G, Gumpert CH (2018). The development of an internet-based treatment for problem gamblers and concerned significant others: A pilot randomized controlled trial. Journal of Gambling Studies.

[CR55] Oei TPS, Gordon LM (2008). Psychological factors related to gambling abstinence and relapse in members of gamblers anonymous. Journal of Gambling Studies.

[CR56] Pallanti S, DeCaria CM, Grant JE, Urpe M, Hollander E (2005). Reliability and validity of the pathological gambling adaptation of the yale-brown obsessive-compulsive scale (PG-YBOCS). Journal of Gambling Studies.

[CR57] Petry NM, Ginley MK, Rash CJ (2017). A systematic review of treatments for problem gambling. Psychology of Addictive Behaviors.

[CR58] Pfund RA, Whelan JP, Peter SC, Meyers AW (2020). Can a motivational letter increase attendance to psychological treatment for gambling disorder?. Psychological Services.

[CR59] Raudenbush SW, Bryk AS (2002). Hierarchical linear models: Applications and data analysis methods.

[CR60] Ronzitti S, Soldini E, Smith N, Clerici M, Bowden-Jones H (2017). Gambling disorder: Exploring pre-treatment and in-treatment dropout predictors. A UK study. Journal of Gambling Studies.

[CR61] Rossini-Dib D, Fuentes D, Tavares H (2015). A naturalistic study of recovering gamblers: What gets better and when they get better. Psychiatry Research.

[CR62] RStudio Team. (2019). *RStudio: Integrated Development for R*. RStudio Inc. https://www.rstudio.com/.

[CR63] Schilling L, Moritz S, Kriston L, Krieger M, Nagel M (2018). Efficacy of metacognitive training for patients with borderline personality disorder: Preliminary results. Psychiatry Research.

[CR64] Schluter MG, Kim HS, Poole JC, Hodgins DC, McGrath DS, Dobson KS, Traveres H (2019). Gambling-related cognitive distortions mediate the relationship between depression and disordered gambling severity. Addictive Behaviors.

[CR65] Schmidt J, Lamprecht F, Wittmann WW (1989). Zufriedenheit mit der stationären Versorgung. Entwicklung eines Fragebogens und erste Validitätsuntersuchungen. Psychotherapie Psychosomatik Medizinische Psychologie.

[CR66] Schmidt, J., & Wittmann, W. W. (2002). ZUF-8 Fragebogen zur Messung der Patientenzufriedenheit. In E. Brähler, J. Schumacher, & B. Strauß (Eds.), *Diagnostische Verfahren in der Psychotherapie* (pp. 392–396). Hogrefe.

[CR67] Schneider BC, Cludius B, Lutz W, Moritz S, Rubel J (2018). An investigation of module-specific effects of metacognitive training for psychosis. Zeitschrift Für Psychologie.

[CR68] Sharpe L (2002). A reformulated cognitive—behavioral model of problem gambling. A Biopsychosocial Perspective.

[CR69] Slutske WS (2006). Natural recovery and treatment-seeking in pathological gambling: results of two U.S. national surveys. American Journal of Psychiatry.

[CR70] Smith DP, Battersby MW, Pols RG, Harvey PW, Oakes JE, Baigent MF (2015). Predictors of relapse in problem gambling: A prospective cohort study. Journal of Gambling Studies.

[CR71] Stange M, Graydon C, Dixon MJ (2016). “I was that close”: Investigating players’ reactions to losses, wins, and near-misses on scratch cards. Journal of Gambling Studies.

[CR72] Stange M, Graydon C, Dixon MJ (2017). Increased urge to gamble following near-miss outcomes may drive purchasing behaviour in scratch card gambling. Journal of Gambling Studies.

[CR73] Strong DR, Breen RB, Lejuez CW (2004). Using item response theory to examine gambling attitudes and beliefs. Personality and Individual Differences.

[CR74] Suurvali H, Cordingley J, Hodgins DC, Cunningham J (2009). Barriers to seeking help for gambling problems: A review of the empirical literature. Journal of Gambling Studies.

[CR75] Tavares H, Zilberman ML, El-Guebaly N (2003). Are there cognitive and behavioural approaches specific to the treatment of pathological gambling?. Canadian Journal of Psychiatry.

[CR76] Thaller R, Specht S, Künzel J, Braun B (2017). Suchthilfe in Deutschland 2016.

[CR77] Toneatto T (2005). A perspective on problem gambling treatment: issues and challenges. Journal of Gambling Studies.

[CR78] Toneatto T (2016). Single-session interventions for problem gambling may be as effective as longer treatments: Results of a randomized controlled trial. Addictive Behaviors.

[CR79] Weinstock J, April LM, Kallmi S (2017). Is subclinical gambling really subclinical?. Addictive Behaviors.

[CR80] Williams JD, Pulford J, Bellringer M, Abbott M (2010). Recruiting gamblers from the general population for research purposes: Outcomes from two contrasting approaches. International Journal of Mental Health and Addiction.

[CR81] Williams, R., Volberg, R., & Stevens, R. (2012). *The population prevalence of problem gambling: Methodological influences, standardized rates, jurisdictional differences, and worldwide trends*. Report prepared for the Ontario Problem Gambling Research Centre and the Ontario Ministry of Health and Long Term Care. https://hdl.handle.net/10133/3068.

[CR82] Xu L, Paterson AD, Turpin W, Xu W (2015). Assessment and selection of competing models for zero-inflated microbiome data. PLoS ONE.

[CR83] Zhang M, Ying J, Song G, Fung DS, Smith H (2018). Gamified cognitive bias modification interventions for psychiatric disorders: Review. JMIR Mental Health.

