# Supplementary material for: Feasibility, Acceptance, and Safety of Metacognitive Training for Problem and Pathological Gamblers (Gambling-MCT): A Pilot Study
Source: J Gambl Stud. 2020 Sep 21;37(2):663–87. doi: 10.1007/s10899-020-09975-w (PMC8144133; doi:10.1007/s10899-020-09975-w)
Supplement: Supplementary file 1 — Supplementary file1 (DOC 715 kb) [file 10899_2020_9975_MOESM1_ESM.doc]

**Supplementary material**

Supplementary material A

Cognitive distortions associated with problem and pathological gambling

|  | Definition | Consequences |
| --- | --- | --- |
| Near-misses | Events that are close to a win (e.g. two of three necessary win symbols) are perceived as a win or win announcement, although they objectively represent loss events | Increased motivation to continue gambling (Clark, Lawrence, Astley-Jones, & Gray, 2009; Stange, Graydon, & Dixon, 2016, 2017)  Neural activity in reward associated brain areas (Clark et al., 2009; Dymond et al., 2014; Sescousse et al., 2016) |
| Illusion of control | Assumption, that the outcome of the game can be influenced through own behaviour or personal luck | Increased motivation to continue gambling (Ladouceur & Sévigny, 2005)  Increased perceived chances of winning, increased satisfaction with the result (Dixon, Larche, Stange, Graydon, & Fugelsang, 2018; Kool, Getz, & Botvick, 2013)  Neural activity in reward associated brain areas (Leotti & Delgado, 2014) |
| Gambler’s fallacy | Assumption that a series of the same event (e.g. "red" in roulette), decreases the probability for this event and increases the probability for the opposite event ("black") | Persistent gambling behaviour (Ayton & Fischer, 2004),  „Chasing“ (Fortune & Goodie, 2012) |
| Illusory correlation | Events are erroneously perceived to be related due to past experience (e.g. superstition) | Increased stakes, gambling time, losses and loss of control (Joukhador, Blaszczynski, & Maccallum, 2004) |
| Memory bias | Selective memory of wins | Increased symptom severity (Navas, Verdejo-García, López-Gómez, Maldonado, & Perales, 2016; Raylu & Oei, 2004) |
| Attributional bias | Internal attribution of wins (e.g. personal skills), external attribution of losses (e.g. slot machines) |

Supplementary material B

*Description of Gambling-MCT modules*

Module 1: Attributional style

The first module of Gambling-MCT targets dysfunctional attributional style. After defining the construct, different attributional styles are discussed with a focus on one-sided attributions (e.g., attributing failure entirely to others). Advantages and disadvantages of one-sided attributions are discussed with participants based on different situations. Participants are encouraged to contemplate attributions that include a variety of causes (i.e., oneself, others, situation/chance). After this, the link between this problem and pathological gambling and one-sided attributions in this population (i.e., internal attribution of wins, external attribution of losses) and their consequences (i.e., continuing gambling, being “trapped” in the game) are discussed. A video exercise demonstrates that certain stimuli (e.g., light, colors, and melodies) of slot machines can further intensify the feeling of being “trapped” in the game. At the end of the module, trainers convey strategies to create alternative, more helpful attributions (e.g., looking at different possible causes, a change of perspective).

Modules 2 and 4: Probabilities I and II

Modules 2 and 4 target gambling-related cognitive distortions on the topic of probability and chance. Both modules start with an introduction to cognitive distortions in general and specific cognitive distortions in problem and pathological gamblers. In module 2, an experiment on anchor heuristics is displayed emphasizing that cognitive distortions occur not only in people with mental disorders but also in nonaffected people. In module 2, two gambling-specific cognitive distortions are addressed: near misses and gambler’s fallacy. In module 4, the illusion of control and illusory correlations are demonstrated. Cognitive distortions are uncovered using fun exercises. For example, in module 2 participants are asked to rate different outcomes of a slot machine game (see figure 1). After each exercise, the relevant cognitive distortion is explained and participants are asked to indicate by a hand signal to what extent this gambling-specific cognitive distortion applies to them.

Moreover, in module 2 participants receive information on probabilities and chance in gambling. Again, fun exercises are used. For example, the function of the green zero in roulette in addition to the 18 black and red numbers—to prevent equal winning odds for gamblers and the gambling provider—is discussed.


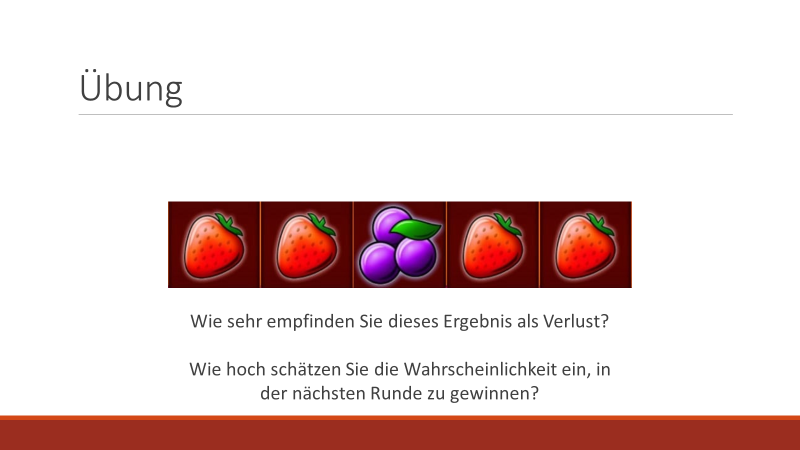


*Figure 1.* Exercise on “near misses.” Translation: “How strongly do you perceive this result as a loss?”; “How do you estimate your probability of winning in the next round?”

At the end of module 4, a quiz is conducted in which various statements on previously learned content from the two probability modules are presented (e.g., “Cognitive distortions are errors in thinking that occur from time to time in almost all people”). The group is asked to discuss whether each statement is true or false.

Module 3: Self-esteem and mood

At the beginning of the third module, participants are asked to come up with a definition of “self-esteem” and discuss characteristics of people with high vs. low self-esteem. Using the metaphor of a set of shelves, different sources of self-esteem (e.g. relationships, sports, profession) are explored. Participants are encouraged to define their personal strengths and write them down in a list they can read and expand regularly.

Afterwards, obstacles to the visualization of strengths, such as unhelpful assumptions (e.g., “Complimenting yourself is rude”) or unfair comparisons with other people, are discussed. Participants are encouraged to consider when and why they unfairly compare themselves to other people.

Next, the connection between thoughts, feelings, and behavior is explained, and it is made clear that low self-esteem promotes a negative state of mind, which in turn can trigger or intensify the urge to gamble. Then, the short-term and long-term consequences of gambling on mood and self-esteem are illustrated and discussed. Participants are instructed to positively influence this connection by changing their behavior (upward spiral).

At the end of the module, participants receive tips on ways to improve their mood and self-esteem (e.g., implementing positive activities, engaging in physical exercise, and keeping a positivity journal).

Module 5: Memory

Module 5 starts with an introduction to memory capacity and its limits. This is followed by an exercise on memory errors in which participants are shown various detailed images and are asked to recollect what was shown in the image (see Figure 2). Details that are not shown in the picture but would fit very well in the scene are often erroneously remembered.

It is emphasized that memory errors can also occur in everyday life (e.g., childhood memories, confusing situations).


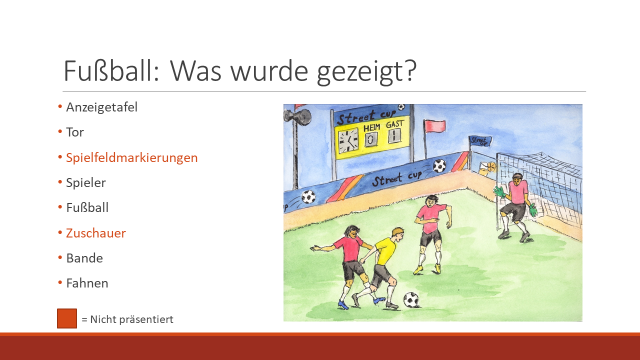


*Figure 2.* Solution of memory exercise. Translation: “Soccer: What was shown in the picture?”; “Scoreboard, goal, field markings, players, ball, spectators, advertisement, flags”; “orange = not presented”

Following this, memory errors in problem and pathological gamblers (i.e., selective memory; wins are remembered better and more vividly than losses) are discussed. Next, subjectivity in memory and influences of mood on memory (e.g., the Pollyanna effect: the tendency of people to remember positive, pleasant things better than negative ones) are discussed. Finally, due to the high prevalence of depressive symptoms in problem and pathological gamblers, the module deals with the depression-related cognitive distortion “mental filter,” which is the tendency to focus on a single negative detail and ignore the positive aspects of a situation.

Participants are encouraged to make positive, more helpful appraisals of situations, which are usually followed by positive consequences.

Module 6: Gambling urge

After defining the construct “gambling urge,” potential triggers for the gambling urge are collected via group discussion. Moreover, positive short-term and negative long-term consequences of gambling and their consequences are discussed (i.e., if a trigger is always responded to in the same way because of positive short-term consequences of this behavior, the behavior becomes an automatic habit). An exercise allows participants to practice identifying and analyzing triggers and the positive and negative consequences of gambling situations using a method called functional analysis. Afterwards, the trainers impart strategies on how to break this automatic pattern, including alternative coping strategies for dealing with triggers (e.g., learning new behaviors and ways of coping, stimulus control) and an acute gambling urge (e.g., pursuing distracting activities, saying helpful sentences to oneself). The module ends with a mindfulness exercise.

Module 7: Debt regulation

In this module, participants learn how to deal with the often subtly accumulating debt caused by their gambling behavior. The process of debt accumulation is illustrated by a downward spiral. Initial small losses of money become bigger and bigger, measures to raise money become more extreme, and the pressure to chase the money back becomes stronger. Trainers impart strategies to stop this downward spiral. These measures can be divided into three main groups: stopping gambling (e.g., house bans in casinos and gambling halls), changing the handling of money (e.g., entrusting somebody with money management, budget, and expenditure protocols), and consolidation of debts and debt counseling. An upward spiral is used to illustrate how these measures may lead to regaining financial control.

Module 8: Relapse prevention

Module 8 includes psychoeducation regarding relapses and the need for self-determined relapse prevention by problem and pathological gamblers. This includes the identification of personal triggers (e.g., stress, negative or positive feelings); physical, emotional, mental, and behavioral warning signs of a relapse; and an appropriate way to react to these signs. Various behaviors are discussed, such as the implementation of positive activities, improving sleep quality, and relaxation. In addition, participants are instructed to develop an emergency plan with a list of positive activities and telephone numbers of emergency contacts as well as measures to reduce relapse-evoking circumstances (e.g., avoiding certain places and persons, temporarily not carrying cash).

Furthermore, the module addresses comorbid, relapse-promoting depressive symptoms. In accordance with MCT’s depathologizing approach, it is initially stated that negative mood and low self-esteem can also occur in healthy individuals. Depressive symptoms and their occurrence in problem and pathological gamblers are presented as a consequence or a precursor of gambling symptoms. Moreover, the module deals with the depression-related cognitive distortion “overgeneralization.”

Supplementary material C

*Equations of linear-mixed models*

The equations we used for calculation of linear-mixed-models are described below. We conducted models (changes over time as well as within-session changes) for all twelve items of the questionnaire on within-session adverse events. The items are referred to as variables or dependent variables in this supplement.


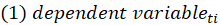
=
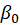
 +
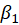
*
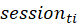
 + [
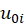
 +
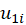
 +
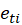
]

First, we calculated changes in dependent variables over the treatment period. In this equation, the dependent variable reflects the variable score of patient *i* for each time point *t*. The mean score of all subjects for the variable before and after each session is depicted by
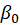
. The slope (
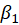
) represents the mean pre-post difference score for each session. The time variable (
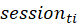
) was centered at 1 as in this analysis variable changes from the first to the last module were computed. Lastly, two variables reflecting the variance between participants in pre-treatment variable score (
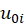
) and the variance of individual pre-post difference score (
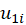
) as well as the module-specific error term (
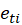
) were included in the model equation.

(2)
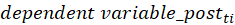
=
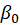
 +
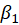
*
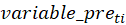
 +
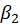
*
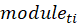
 + [
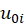
 +
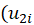
)+
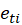
]

In the second analysis we calculated within-session changes for each module (
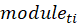
). Each session, in which subject *i* attained the investigated module was coded with a 1 while all other seven time points were coded with a 0. The term
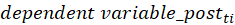
 represents the variable score of patient *i* after each session *t*. Again,
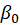
 stands for the average variable score, except in this model, the average score is calculated for time points in which the respective module was not implemented. The first slope control for variance in pre-session variable scores (
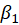
*
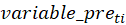
), while the term
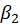
*
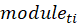
 represents the mean difference variable score after the session the investigated module was conducted comparing it to all other time points the respective module was not conducted. Like in the previous model,
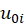
 reflects between-subject variance pre-treatment. Within-subject variance (random effects) was predicted by the term
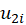
.The expression
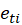
 once more stands for the session-specific error term.

Supplementary material D

| Models that analyzed the changes from first to last treatment session. | | |
| --- | --- | --- |
| Variable | AIC of the random intercept model | AIC of the random intercept, random slope model |
| Gambling thoughts | 247.7 | 251.5 |
| Control over gambling | 285.8 | 289.8 |
| Efforts to resist thoughts | 265.5 | 267.3 |
| Impairment due to gambling thoughts | 259.5 | 262.4 |
| Abstinence intention | 183.9 | 187.8 |
| Abstinence confidence | 160.0 | 152.1 |
| Financial trigger | 259.4 | 254.6 |
| Sadness | 261.9 | 261.8 |
| Restlessness | 281.2 | 284.4 |
| Positive associations | 225.3 | 227.8 |
| Illusion of control | 228.8 | 224.3 |
| Chasing | 183.0 | 170.6 |

| Models that analyzed within-session changes. | | |
| --- | --- | --- |
| Variable | AIC of the random intercept model | AIC of the random intercept, random slope model |
| Gambling thoughts  Modul 1  Modul 2  Modul 3  Modul 4  Modul 5  Modul 6  Modul 7  Modul 8 | 244.0  235.0  250.4  251.0  249.6  250.9  251.0  250.9 | 247.0  206.1  248.9  254.3  251.5  254.7  254.4  253.5 |
| Control over gambling  Modul 1  Modul 2  Modul 3  Modul 4  Modul 5  Modul 6  Modul 7  Modul 8 | 279.5  274.1  277.2  279.1  279.7  279.4  278.2  278.4 | 282.6  277.9  281.2  282.9  283.7  281.8  282.2  276.8 |
| Efforts to resist thoughts  Modul 1  Modul 2  Modul 3  Modul 4  Modul 5  Modul 6  Modul 7  Modul 8 | 265.6  260.4  265.2  265.6  265.1  265.6  265.3  260.8 | 268.5  252.7  267.8  269.6  266.5  267.6  267.4  260.0 |
| Impairment due to gambling thoughts  Modul 1  Modul 2  Modul 3  Modul 4  Modul 5  Modul 6  Modul 7  Modul 8 | 241.9  252.8  252.7  253.0  252.0  253.0  253.0  252.3 | 239.8  254.6  256.0  256.7  256.0  255.6  257.0  255.2 |
| Abstinence intention  Modul 1  Modul 2  Modul 3  Modul 4  Modul 5  Modul 6  Modul 7  Modul 8 | 26.3  26.8  26.4  26.7  26.8  26.7  26.7  26.8 | 20.7  25.1  25.3  27.3  27.8  26.9  30.5  27.1 |
| Abstinence confidence  Modul 1  Modul 2  Modul 3  Modul 4  Modul 5  Modul 6  Modul 7  Modul 8 | 112.3  112.3  111.7  111.5  111.7  112.4  112.1  112.4 | 112.2  108.9  109.0  109.2  115.3  116.4  115.5  108.3 |
| Financial trigger  Modul 1  Modul 2  Modul 3  Modul 4  Modul 5  Modul 6  Modul 7  Modul 8 | 245.2  245.1  249.5  249.5  249.5  249.2  248.0  248.7 | 232.5  249.0  251.2  252.8  253.4  253.2  249.1  251.1 |
| Sadness  Modul 1  Modul 2  Modul 3  Modul 4  Modul 5  Modul 6  Modul 7  Modul 8 | 215.4  216.1  212.1  215.5  214.4  216.0  215.5  215.9 | 217.7  218.0  215.1  219.5  218.2  220.0  219.5  217.2 |
| Restlessness  Modul 1  Modul 2  Modul 3  Modul 4  Modul 5  Modul 6  Modul 7  Modul 8 | 235.0  235.2  234.8  231.8  234.8  235.3  235.3  235.2 | 237.5  231.1  234.8  234.4  238.7  239.2  239.2  238.7 |
| Positive associations  Modul 1  Modul 2  Modul 3  Modul 4  Modul 5  Modul 6  Modul 7  Modul 8 | 184.5  176.1  183.0  182.3  184.4  183.4  184.8  184.7 | 177.2  168.8  181.1  186.1  188.8  185.5  188.8  188.7 |
| Illusion of control  Modul 1  Modul 2  Modul 3  Modul 4  Modul 5  Modul 6  Modul 7  Modul 8 | 163.3  158.3  161.0  163.0  163.4  163.4  163.4  163.4 | 158.4  115.5  149.5  166.1  166.2  165.9  164.9  166.1 |
| Chasing  Modul 1  Modul 2  Modul 3  Modul 4  Modul 5  Modul 6  Modul 7  Modul 8 | 157.7  152.5  156.1  157.5  158.0  157.5  157.7  156.3 | 158.5  145.7  158.2  161.1  161.9  159.8  160.4  155.8 |
